# Spatial pattern analysis of nuclear migration in remodelled muscles during *Drosophila* metamorphosis

**DOI:** 10.1186/s12859-017-1739-0

**Published:** 2017-07-10

**Authors:** Kuleesha, Lin Feng, Martin Wasser

**Affiliations:** 1 School of Computer Science and Engineering, Nanyang Technological University, Singapore, Singapore; 2 Imaging Informatics Division, Bioinformatics Institute (BII), Agency for Science, Technology and Research (A*STAR), Singapore, Singapore; 3 BioImagingMW, Block 28D Dover Crescent #31-73, Singapore, 134028 Singapore

**Keywords:** Drosophila metamorphosis, Myonuclear localization, Nuclear segmentation, Nuclear tracking, Classification, Feature generation

## Abstract

**Background:**

Many human muscle wasting diseases are associated with abnormal nuclear localization. During metamorphosis in *Drosophila melanogaster*, multi-nucleated larval dorsal abdominal muscles either undergo cell death or are remodeled to temporary adult muscles. Muscle remodeling is associated with anti-polar nuclear migration and atrophy during early pupation followed by polar migration and muscle growth during late pupation. Muscle remodeling is a useful model to study genes involved in myonuclear migration. Previously, we showed that loss of Cathepsin-L inhibited anti-polar movements, while knockdown of autophagy-related genes affected nuclear positioning along the medial axis in late metamorphosis.

**Results:**

To compare the phenotypic effects of gene perturbations on nuclear migration more objectively, we developed new descriptors of myonuclear distribution. To obtain nuclear pattern features, we designed an algorithm to detect and track nuclear regions inside live muscles. Nuclear tracks were used to distinguish between fast moving nuclei associated with fragments of dead muscles (sarcolytes) and slow-moving nuclei inside remodelled muscles. Nuclear spatial pattern features, such as longitudinal (lonNS) and lateral nuclear spread (latNS), allowed us to compare nuclear migration during muscle remodelling in different genetic backgrounds. Anti-polar migration leads to a lonNS decrease. As expected, lack of myonuclear migration caused by the loss of *Cp1* was correlated with a significantly lower lonNS decrease. Unexpectedly, the decrease in lonNS was significantly enhanced by *Atg9*, *Atg5* and *Atg18* silencing, indicating that the loss of autophagy promotes the migration and clustering of nuclei. Loss of autophagy also caused a scattering of nuclei along the lateral axis, leading to a two-row as opposed to single row distribution in control muscles. Increased latNS resulting from knockdown of *Atg9* and *Atg18* was correlated with increased muscle diameter, suggesting that the wider muscle fibre promotes lateral displacement of nuclei from the medial axis during polar migration.

**Conclusions:**

We developed new nuclear features to characterize the dynamics of nuclear distribution in time-lapse images of *Drosophila* metamorphosis. Image quantification improved our understanding of phenotypic abnormalities in nuclear distribution resulting from gene perturbations. Therefore, in vivo imaging and quantitative image analysis of *Drosophila* metamorphosis promise to provide novel insights into the relationship between muscle wasting and myonuclear positioning.

**Electronic supplementary material:**

The online version of this article (doi:10.1186/s12859-017-1739-0) contains supplementary material, which is available to authorized users.

## Background

Skeletal muscle fibres are large multinucleated cells. Nuclei inside of muscle cells (myonuclei) are thought to be positioned such that the local nuclear-cytoplasmic ratio remains constant [1]. One reason for this behaviour is that a nucleus can only support a fixed volume of cytoplasm, called a myonuclear domain (MND) [2], due to the limited distance that proteins can be transported inside cells [3]. Therefore, in healthy muscles, nuclei are expected to be evenly distributed. A quantitative study on the spatial distribution of nuclei in mice has confirmed that the myonuclei are not randomly distributed and are arranged in a row-like formation, indicating that the nuclei could be repelling each other to minimize the transport distance [4]. Unlike healthy muscles, several studies have revealed abnormal MND sizes in hypertrophic and atrophied muscles [5, 6]. Centrally positioned nuclei have been observed in many muscle disorders, including central nuclear myopathies [7] and muscular dystrophy [8, 9]. Previous studies have shown that nuclear envelope proteins play a role in regulating nuclei positioning [10, 11]. In a study on *Drosophila* larvae, the KASH mutants showed impaired locomotion and aggregation of the myonuclei [12]. Loss of JNK signalling also caused clustering of nuclei and large regions in muscles devoid of nuclei [13]. Despite numerous studies on nuclear positioning, its role in muscle function remains unclear.

**Fig. 1:**
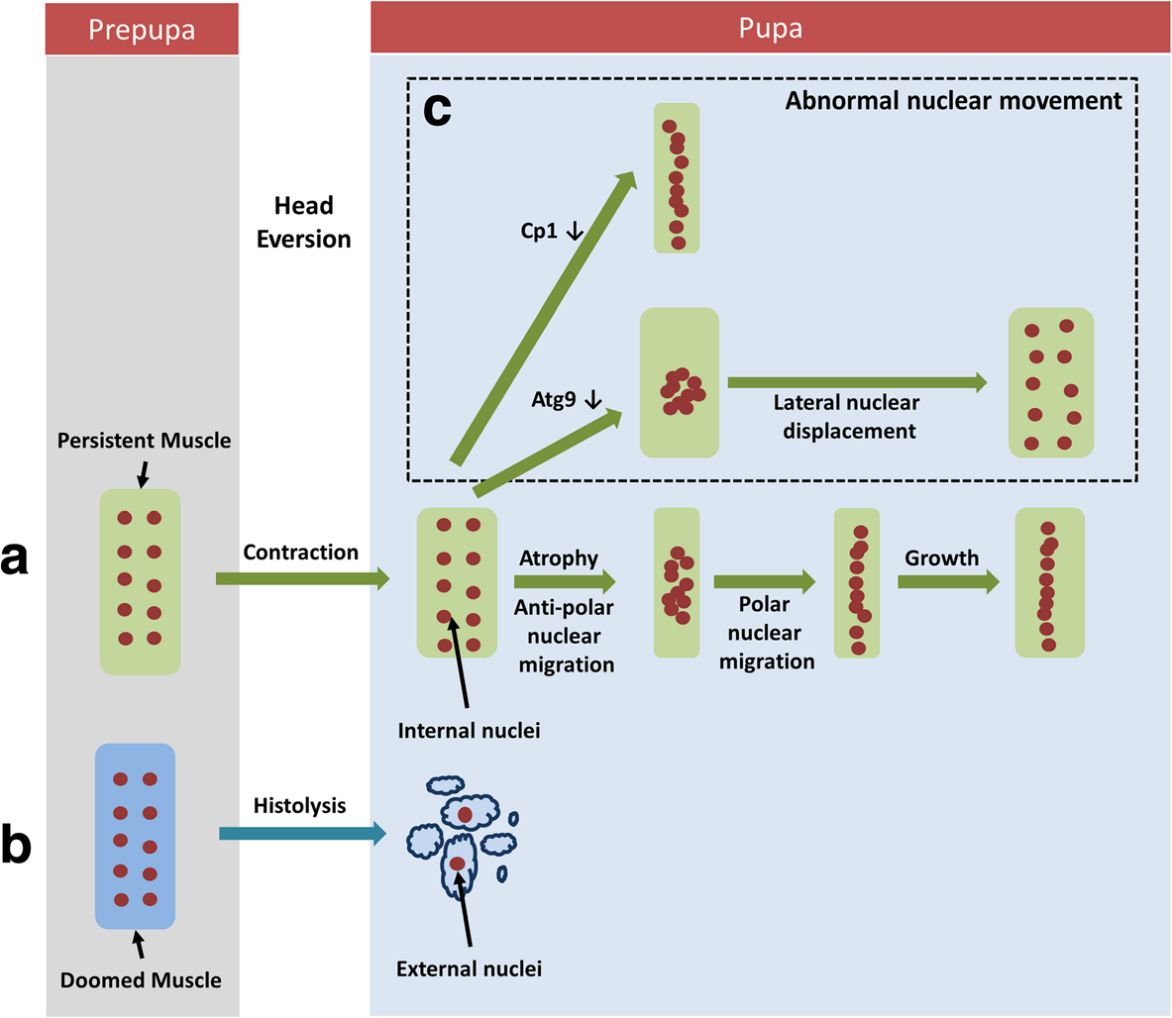
Schematic diagram explaining different stages of nuclear localization. **a** In persistent muscles, the nuclear positioning transitions from an initial two-row like formation in prepupae to a clustered distribution in mid-pupation, and lastly a one row formation in late pupation. **b** A sub-set of DIOM undergo histolysis and create muscle debris with nuclei inside them. These nuclei form the external nuclei. **c** Knockdown of *Cp1* and *Atgs* affect the myonuclear distribution

Previously, we reported that, during Drosophila metamorphosis, which last 4 to 5 days, the nuclei in abdominal dorsal internal oblique muscles (DIOM), also referred to as persistent muscles show changes in myonuclear distribution [14]. In larval and prepupal stages, nuclei show an even distribution within the muscle fibres (Fig. 1). After head eversion (HE), taking place approximately 12 h after puparium formation, most skeletal muscles undergo programmed cell death and become fragmented, while persistent muscles survive into adulthood. We will refer to nuclei inside the persistent muscles as internal nuclei and nuclei inside sarcolytes (muscle fragments) as external nuclei. In the first 2 days of pupation after HE, persistent muscles undergo atrophy and their nuclei start migrating in an anti-polar fashion towards the centre of the muscle. At mid-pupation, the direction of myonuclear migration reverses and the nuclei move back to the poles while positioning themselves along the medial axis of muscles. While the muscle diameter increases in late pupation, the nuclei remain anchored in a single-row formation along the medial axis. Myonuclear migration was also reported to occur in early myogenesis when mouse myoblasts fuse with myotubes [15] and *Drosophila* embryonic myoblasts fuse with founder cells [16], suggesting that muscle remodelling could be interpreted as dedifferentiation of mature muscles into a myotube-like state. In a pilot forward genetics RNAi screen, we also identified the first genes that play roles in the migration and positioning of nuclei in remodelled muscles [14]. Silencing of *Cp1*, the gene encoding the homolog of the lysosomal proteases Cathpesin-L inhibited anti-polar migration in early migration. Knockdown of several autophagy-related genes (*Atg5, Atg9, Atg12. Atg18*) resulted in scattering of nuclei along the lateral axis in late metamorphosis, giving the appearance of a double-row formation (Fig. 1c, Additional file 1: Figures S1 & S2) [14]. To better understand myonuclear distribution and compare the phenotypes resulting from genetic perturbations more objectively, new methods for the quantitative analysis of nuclear migration and localization are required. Spatial pattern analysis has been used to investigate the sub–cellular localization of centromeres [17], nuclei in multi-nucleated muscles [4], and nuclei in *Drosophila* embryos [18].

In this paper, we present our spatial pattern analysis algorithm to study the effects of genetic perturbations on the distribution of nuclei in remodelled *Drosophila* muscles during metamorphosis. Our method consists of two parts. First, we detect and track nuclei inside remodelled muscles expressing Mhc-tau-GFP and Histone-mKO to label cytoplasm and nuclei in two different colors. Since we analyse 2D projections of 3D image stacks, we need to classify nuclei inside muscle regions into slow-moving internal and fast-moving external nuclei. We demonstrate high accuracy for the segmentation, tracking and classification steps. Second, we calculate static and dynamic spatial pattern features of slow-moving nuclei corresponding to remodelled muscles. The longitudinal and lateral nuclear spreads and their changes over time helped us detect significant phenotypic variations between different genotypes that were not discernible by eyeballing. As such, quantitative analysis of nuclear migration and localization will improve the depth of phenotypic profiling in time-lapse image analysis.

## Methods

We used the UAS-GAL4 system to achieve targeted expression of fluorescent proteins and shRNA (small hairpin) in muscles. Muscle cytoplasm and nuclei were labelled using MHC-tau-GFP [19] and UAS-Histone 2Av-mKO [20], respectively. Mef2-GAL4 was used as a muscle specific driver [21]. All UAS-shRNA (small hairpin) transgenic lines were obtained from Transgenic RNAi Project (TRiP) collection [22]. In our experiment, we crossed female of reporter line *MHC-tau-GFP/FM7-GFP*; *Mef2-GAL4, UAS-histone-mKO/TM6B Tb* with male of *UAS-GeneX-RNAi* lines. We examined muscles of non-tubby progeny with genotype *MHC-tau-GFP/+; Mef2-GAL4, UAS-histone-mKO/UAS-GeneX-RNAi.* In our study, we used RNAi lines of the following genes: *Chromator* (Control, Bloomington Stock id: B-36084), *Atg9* (B-34901), *Atg18* (B-34714), *Atg5* (B-34899), *Atg12* (B-34675) and *Cp1* (B-32932). The cross was done at 25 °C.

The protocol for sample preparation and microscopy has been previously described [23, 24]. Line scanning Zeiss LSM 5 Live microscope was used to perform live imaging of *Drosophila* pupae. 20–30 pupae were imaged simultaneously using multi location imaging feature of line scanning microscope. We performed imaging for a duration of 4–5 days. Images were collected at an interval of 30 min. We also collected images of pupae at multiple focal planes. Two color channels were imaged: channel 1 with an excitation laser of 488 nm, band path filter (BP) 500–525; and channel 2 with 532 nm laser line, BP 560–675. The image acquisition was done with the following settings: 10× magnification (EC Plan-Neofluar 10×/0.30 M27), pin hole size of 16.6 μm and frame speed of 2 FPS. The images were of size 1024 × 1024 pixels, and the physical size of each pixel was 1.25 × 1.25 × 11.08 μm. The confocal imaging generated LSM files for each time point. LSM files of every time point of a pupae were concatenated into an ICS file using custom software [25]. For time series analysis of images, the 3D stacks of ICS files were converted into their 2D projections using the maximum intensity projection (MIP) method. The final result was a multi-tiff file in which each image represents a time point. This multi-tiff file was used as an input for nuclear spatial pattern analysis.

### Nuclear spatial pattern analysis pipeline

**Fig. 2:**
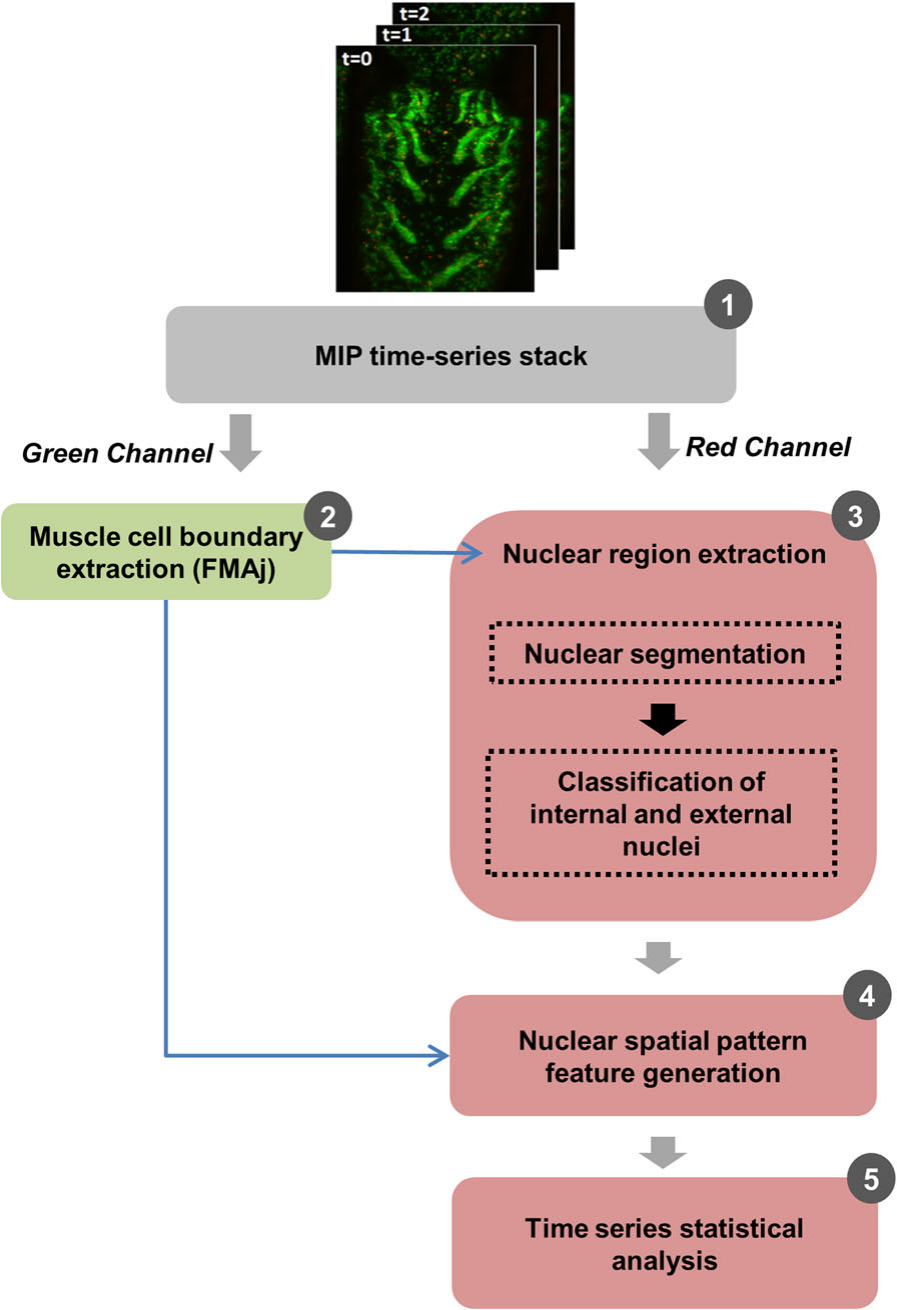
Workflow of the nuclear spatial pattern analysis pipeline. **1** The pipeline takes a multi-tiff time series stack as input. **2** The muscle cell boundary is extracted using FMAj tool. **3** The colour channel which contains nuclei is segmented to obtain the nuclear regions. The muscle boundary is used to remove nuclei which lie outside the muscle cell. The external nuclei (present inside the fragments of dead muscle) are identified and removed from the segmentation results to avoid incorrect feature calculations. **4** & **5** Nuclear spatial pattern features are calculated using the extracted nuclear regions and these features are used for time series statistical analysis of the myonuclear distributions and localization during metamorphosis

A schematic diagram of the nuclear spatial pattern analysis has been shown in Fig. 2.

#### Nuclear region extraction inside persistent muscles

**Fig. 3:**
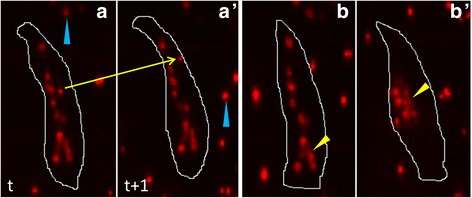
**a** Examples of internal and external nuclei. **b** Examples of nuclear clustering. **a** The *arrow* show the movement of an external nucleus over a muscle cell between time points *t* (**a**) and *t* + 1 (**a’**). The rest of the slow moving nuclei inside the muscle cell are internal nuclei. The *cyan* arrowheads show external nuclei located outside muscle cells. **b** & **b’** The yellow arrowheads show the clumping of nuclei in two different muscle cells

Apart from the nuclei inside the persistent muscles (internal nuclei), the nuclei inside dead muscle fragments (external nuclei) are also present in the pupa abdomen, as shown in Fig. 3a, a’. To calculate nuclear features, we require only the region occupied by internal nuclei. Therefore, after nuclear segmentation, removal of external nuclei from segmented nuclear regions is an important step in myonuclear spatial pattern analysis. The external nuclei which are located outside the persistent muscles can be removed easily using muscle boundary. However, it is difficult to remove external nuclei which appear to be inside persistent muscles due to 2D projection; while they are actually located above or below the persistent muscles. We could not use 3D images for removal of external nuclei due to low z resolution. To tackle these problems, we designed a new algorithm for extracting regions occupied by internal nuclei. Key techniques of the algorithm are:

1. Muscle Segmentation

As mentioned before, we require the muscle boundary to remove external nuclei located outside persistent muscles. The color channel of input stack containing muscle cells is used for segmentation. The muscle boundaries are obtained by using an imageJ based muscle analysis tool, FMAj [23]. We also extract morphological features from muscle boundary to understand the relationship between nuclear distribution and muscle mass change.

2) Nuclear Segmentation

Due to low resolution of images, it is difficult to extract boundaries of nuclei when they are close to each other. For example, a clustered group of nuclei appear as a large blob of bright fluorescence, as shown in Fig. 3b, b’. For this reason, instead of detecting each nucleus, we extract regions where nuclei are located. Each region can contain one nucleus or multiple nuclei. Nuclear segmentation is used to detect myonuclei. To avoid incorrect segmentation due to imaging noise, we first smoothen the image using a bi-exponential edge preserving smoother (BEEPS) [26]. This technique smoothens the high intensity spots near the muscle boundary which occur due to dual channel imaging, while retaining the edge information of nuclei.

**Fig. 4:**
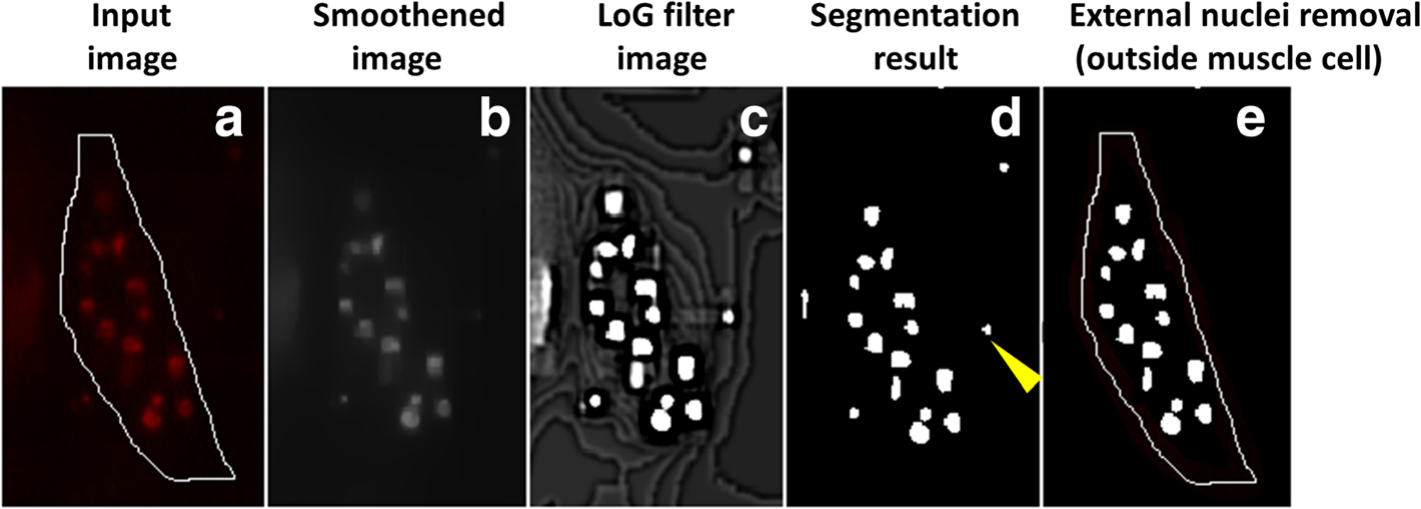
Results of different stages of myonuclear segmentation. The figure shows the input image for nuclear segmentation (**a**) and the results of image smoothening using BEEPS (**b**), LoG filtering (**c**) and thresholding (**d**). The bright spots in (**d**) are the regions occupied by nuclei. Using muscle cell boundary, we remove the external nuclei located outside muscle cells (*yellow arrowhead*) as shown in (**e**). The white contour is the muscle cell boundary

For segmentation of nuclei, we use the negative Laplacian of Gaussian (LoG) filter based scheme [27]. The LoG filter has been used previously to find dark circular spots of radius σ surrounded by bright backgrounds [28, 29]. The general idea is that after Gaussian blurring, the intensity distribution of a nuclei form a smooth ridge and the LoG filter can locate the nuclei by detecting the peak point of these ridges. We use a negative LoG kernel (i.e., −*L*_*σ*_) to enhance bright nuclei surrounded by a dark background. Here, σ = 6 which is the average radius of myonuclei in our dataset, is used. In our images, the LoG filter increases the intensity of the regions where nuclei are present. We achieve nuclear segmentation by applying binary thresholding to the filtered image. Any pixel of intensity 255 was labelled as nuclear region whereas the rest of the pixels were treated as background. Figure 4 shows the results of different stages of nuclear segmentation. Smoothening of image results in decrement in the number of false positives during segmentation (Table 1). Also, by using the LoG filter as pre-processing step, we improve the segmentation of nuclei which have relatively low intensity as compared to the others.

**Table 1:** Nuclear segmentation performance evaluation

	True positive	False positive	False negative	False positive rate	False negative rate
Otsu thresholding without LoG filter	846	0	108	0	0.11
LoG based segmentation with BEEPS smoothening	1180	29	0	0.024	0
LoG based segmentation without BEEPS smoothening	1180	334	0	0.221	0

Since we only want the nuclei present inside the muscle cells for analysis; after obtaining the segmentation results, the external nuclei located outside the muscle boundary are removed, as shown in Fig. 4e. In addition, we have to remove the external nuclei that appear inside muscles as a result of overlapping in image projections. We will describe the classification technique used for removing external nuclei in the next section.

3) Nuclear Tracking and Classification

In order to remove the external nuclei from the segmentation results, we designed a new methodology to classify external and internal nuclei based on their movements. There is a significant difference in the movement of these two types of nuclei. The external nuclei move faster (25–37 μm/h) than internal nuclei (0–12 μm/h). In our classification methodology, we use such a motion characteristic to differentiate between external and internal nuclei. First, we obtain the tracks of nuclei based on a proximity criterion and then classify these tracks on the basis of a cost function derived from nuclear movement in each track.

Various studies were done on tracking nuclei previously [30–32]. However, these problems were customized for tracking nuclei during cell division. On the other hand muscles have multiple nuclei and they don’t undergo division. Combined with various issues in our dataset like indistinguishable nuclei due to their adhering to each other, missing nuclei in many time points due to movement of muscles during imaging, etc.; makes it a unique problem which cannot use the previous nuclei tracking techniques. In the followings, we discuss our approach on nuclear tracking and classification in detail.

##### Step 1: Generating tracks of nuclei

**Fig. 5:**
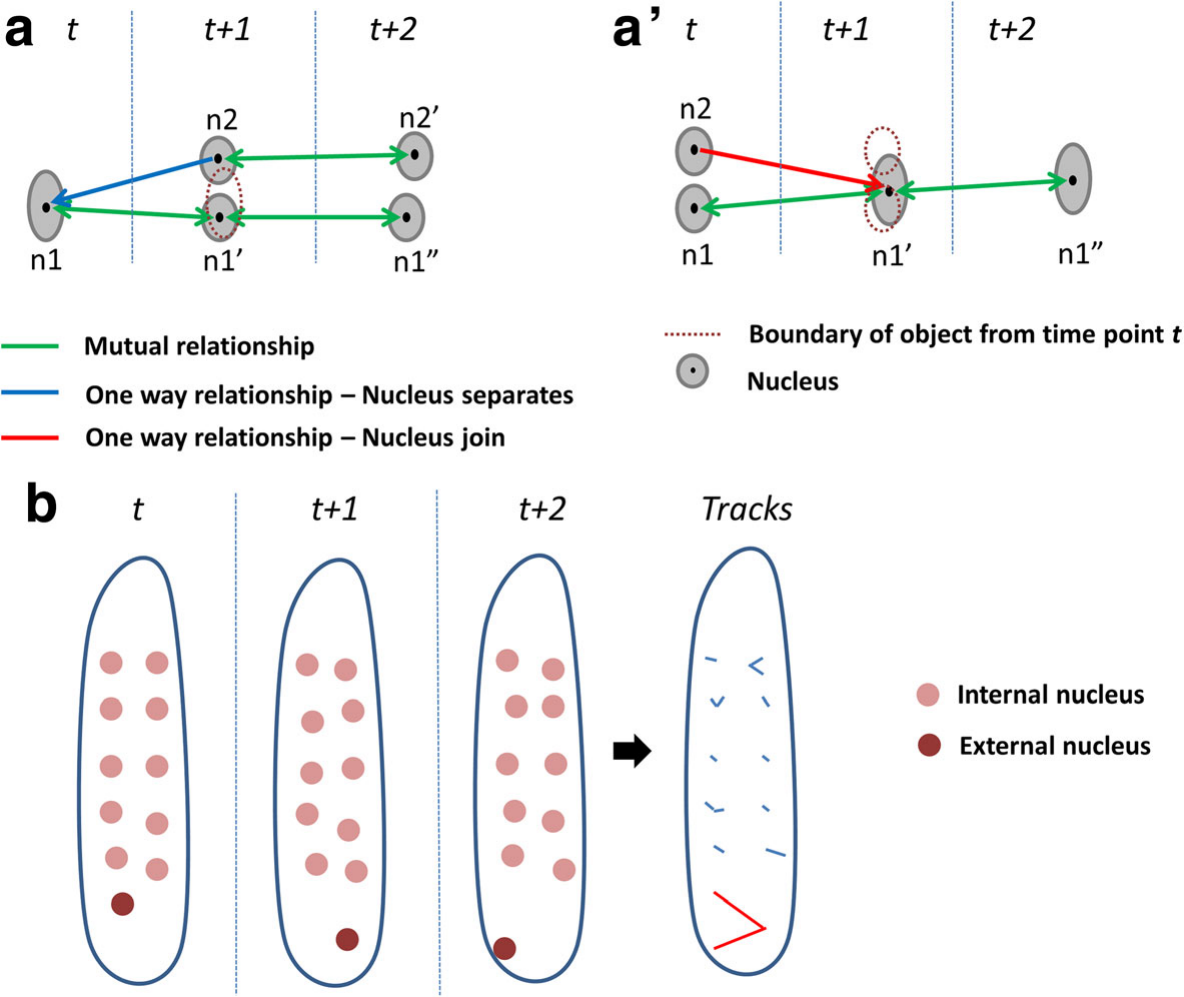
Nuclear tracking. **a** Two nuclei which formed a clump *n1* at time point *t*, separate at *t* + 1. *n1* and *n1’* are mutually closest to each other; therefore *n1’* is added into the track containing *n1*. Whereas, *n2* starts a new track. **a’** Two internal nuclei *n1* and *n2* at *t* form a clump at *t* + 1. Since *n2* does not have mutual closeness to *n1’*; the track containing *n2* terminates at *t*. Whereas, *n1* forms a track which contains *n1’* and *n1”*. **b** The diagram shows an example of tracks generated by nuclei movement between three time points. The movement of external nuclei is larger than the internal nuclei

First, we use connected component analysis on the nuclear regions extracted in previous section, to detect and label blobs [33, 34]. Each blob can contain one nucleus or multiple nuclei. We use these labelled nuclei to generate tracks. However, in our dataset, it is impossible to track a nucleus from beginning to the end of time series. As mentioned before, at certain time points the nuclei are so close to each other that it is difficult to distinguish them, and due to this issue, we get incomplete tracks of nuclei. Therefore, instead of trying to track nuclei throughout the development, we generate multiple smaller tracks. For example, we are tracking a nuclei *n*_*1*_ and the track number is *k*_*1*_. At a time point *T* = *t*, *n*_*1*_ comes close to another nucleus/group of nuclei and form a large clump of high intensity (individual nucleus not visible). At time point *T* = *t* + 1, a nucleus separates from the group of nuclei. However, we are not sure whether it is nucleus *n*_*1*_ or other nucleus from the group. In order to avoid this discrepancy, we call this separated nucleus *n*_*2*_ and create a new track *k*_*2*_. In this manner, we create multiple tracks of the same nucleus over a period of time. The tracks are created based on the nearest neighbour approach. For a nucleus at time point *t*, its nearest neighbour at time point *t* + 1 is found using the minimum distance between centroids of nuclei as criterion. Similarly, for each nucleus at time point *t* + 1, its nearest neighbour at time point *t* is found. We observed three types of relationships between nuclei in adjacent time points as shown in Fig. 5a, a’.

Case I: Nucleus *n1* from time point *t* is the nearest neighbour of nucleus *n1’* from time point *t* + 1 and nucleus *n1’* from time point *t* + 1 is the nearest neighbour of nucleus *n1* from time point *t*. Therefore, they have a mutual relationship, indicating that these two nuclei are the same.

Case II: Nucleus *n1* from time point *t* is the nearest neighbour of nucleus *n2* from time point *t* + 1, but nucleus *n2* is not the nearest neighbour to nucleus *n1*. This indicates that *n1* is a group of nuclei instead of single nucleus and *n2* is a nucleus that has broken off from group of nuclei *n1* (Fig. 5a). Here, ‘broken off’ refers to separation of nuclei which are very close to each other.

Case III: This is the opposite of case II. Nucleus *n1’* from time point *t* + 1 is the nearest neighbour to nucleus *n2* from time point *t*; however, nucleus *n1’* is not the nearest neighbour of nucleus *n2* (Fig. 5a’). This would indicate that *n1’* is a group of nuclei that was formed by joining nucleus *n2* with another nucleus. Here, joining refers to two or more nuclei adhering to each other, making them indistinguishable.

Next, we explain the approach used to generate tracks from the relationships between nuclei in adjacent time points. At the first time point of time series stack, every nucleus starts a new track. If a nucleus has mutual relationship with another nucleus from the next time point (Case I), the track continues. However, if a nucleus has one way relationship with a nucleus in the next time point (Case III), its track terminates. New tracks are created when a nucleus does not have a mutual relationship with any nucleus in previous time point (Case II) (Fig. 5a, a’). We represent the tracks in the form of a T by X table; where T is the time point and X is the total number of tracks. It contains the labels of nuclei. Each nucleus in a time point has a unique label. These unique labels are generated by finding connected components in the image (binary image containing nuclear region as 255 and background as 0) and labeling each component/nuclear region. The labels are assigned based on the location of nuclei along the y axis. Therefore, if there is a difference in the sequence of nuclei along y axis in subsequent time points, the same nuclei will have different labels. If external nuclei are also present, the labels of nuclei change. Track 2 in Additional file 1: Table S1 has different labels at many time points for the same nucleus. In the case of track 1, the nucleus is closest to x axis and there are no nuclei in its proximity; therefore its label does not change in subsequent time points.

We are able to track the nuclei accurately when their movement between adjacent time points is close to zero. However, that is not the case throughout pupal development. The movement of muscle cells varies during the development of pupa. Between 12 and 40 h after head eversion, due to fast movement of muscle cells, the distance covered by internal nuclei in adjacent frames is high (>10 pixels/12.4 μm distance between centroids of muscle cell in consecutive time points). Between 40 to 90 h after head eversion, muscles move slowly. In order to correctly track the nuclei between two consecutive time points, we adjust the position of nuclei at one of the time points to compensate the movement of muscle cells. First, we find the amount of displacement muscle cell undergo, by measuring the displacement of its centroid in two consecutive time points. For example, centroid of muscle cell moved *x* pixels horizontally and *y* pixels vertically between time point *t* and *t* + 1. Next, we translate the image at time point *t* horizontally by *x* pixels and vertically by *y* pixels, so that the nuclei at time point *t* and *t* + 1 align with each other. This alignment facilitates the tracking process by reducing the number of incorrect matching of nuclei between consecutive time points.

##### Step 2: Distinguishing internal from external nuclear tracks

The movement of internal nucleus is much slower than the movement of external nucleus. We exploit this property to classify these two types of nuclei. Intuitively, if a track belongs to an external nucleus, then the average movement of nucleus between consecutive frames should be higher as compared to a track which belongs to an internal nucleus. A schematic diagram in Fig. 5b shows the difference between the track of an internal and external nucleus. Therefore, we design a cost function which is an indicator of the nuclei motion. For a track *x* of length *n*_*x*_ which starts at time point *t*_*s*_ and ends at time point *t*_*e*_, the cost function *M*(*x*) is given as

1 $$ M(x)=\frac{1}{n_x}\;{\sum}_{i={t}_s+1}^{i={t}_e}{D}_i\ast \left(1-{O}_i\right) $$

where *D*_*i*_ is the distance between centroid of nuclei in consecutive time points and *O*_*i*_ is the percentage overlap between nuclei in consecutive time points. Higher value of cost function indicates higher possibility of the track belonging to an external nucleus and vice versa. The overlap factor (*O*_*i*_) increases the cost when the overlap between nuclei is low.

**Fig. 6:**
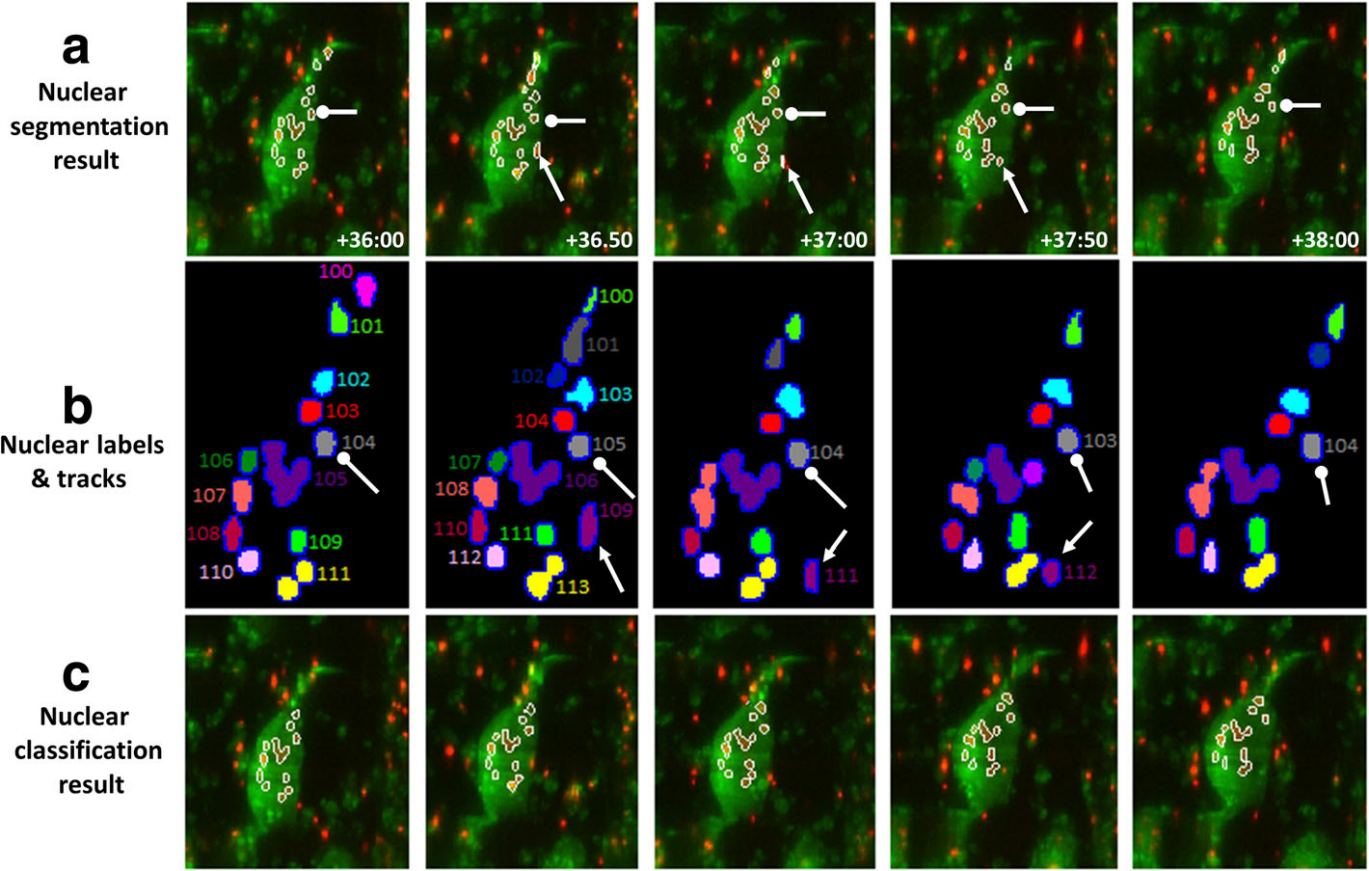
Results of nuclear classification. **a** The images show the result of nuclear segmentation for five time points selected from a pupa expressing *Atg9* RNAi. DIOM muscle from 3rd segment of pupa abdomen was used for this analysis. The segmented nuclei contain both internal and external nuclei. The white arrow and oval arrow indicates an external and internal nucleus. **b** The labels of nuclei at each time point are shown. Nuclei which have same colour in subsequent time points belong to same track. The details of nuclear tracks are shown in Additional file 1: Table S3. **c** The images show results of nuclear classification. The contours of classified external nuclei are removed from the segmentation results

After obtaining the cost function for every track, we classify them based on a cost cut-off. Tracks whose cost function is higher than a threshold λ are classified as tracks of external nuclei. The external nuclei from these tracks are removed from the segmentation results (Fig. 6). Since, the position of nuclei is normalized according to the movements of muscle cells during calculations; the threshold λ should be same for every time series stack. We obtain the value of threshold by performing accuracy tests on training datasets, which will be discussed in the results section.

#### Nuclear spatial pattern feature

During certain time points, it is impossible to identify the location of each nucleus because multiple nuclei adhere to each other and form a large clump of high intensity. In these cases, extraction of nuclear region generates a blob (connected components) which contains many nuclei. For this reason, we cannot use point pattern analysis [18] to study nuclear distribution, but have to design a different approach which uses complete nuclear structure inside muscles as compared to only centroids for nuclear pattern feature generation. We design three new features which quantify different types of nuclear distributions, i.e. nuclear spatial density index, longitudinal nuclear spread and lateral nuclear spread. Before analysing the nuclear distribution, we rearrange the nuclear structure along the straightened medial axis of the muscle cell.

#### Rearrangement of nuclei in straightened muscle cell

**Fig. 7:**
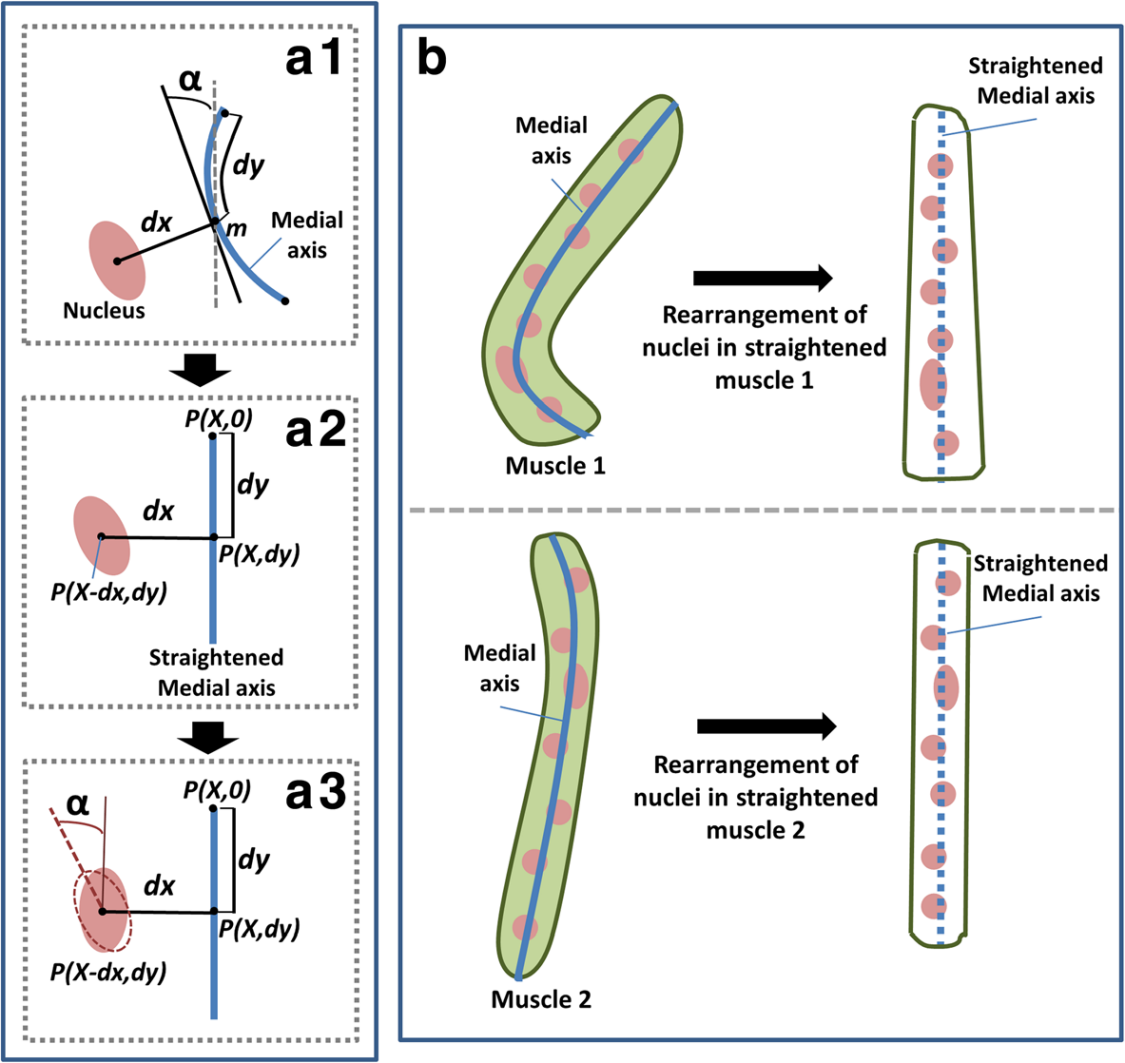
Rearrangement of nuclei in straightened muscle cell for comparing nuclei from different samples. **a1**-**a3** The figure shows various steps involved in rearrangement of a nucleus along straightened muscle medial axis i.e. calculation of new coordinates of nucleus and medial axis (**a1**), translation of nucleus to new coordinate (**a2**) and rotation of nucleus about its centroid (**a3**). **b** Rearranged nuclei along medial axis are shown for two muscles with different shapes

**Fig. 8:**
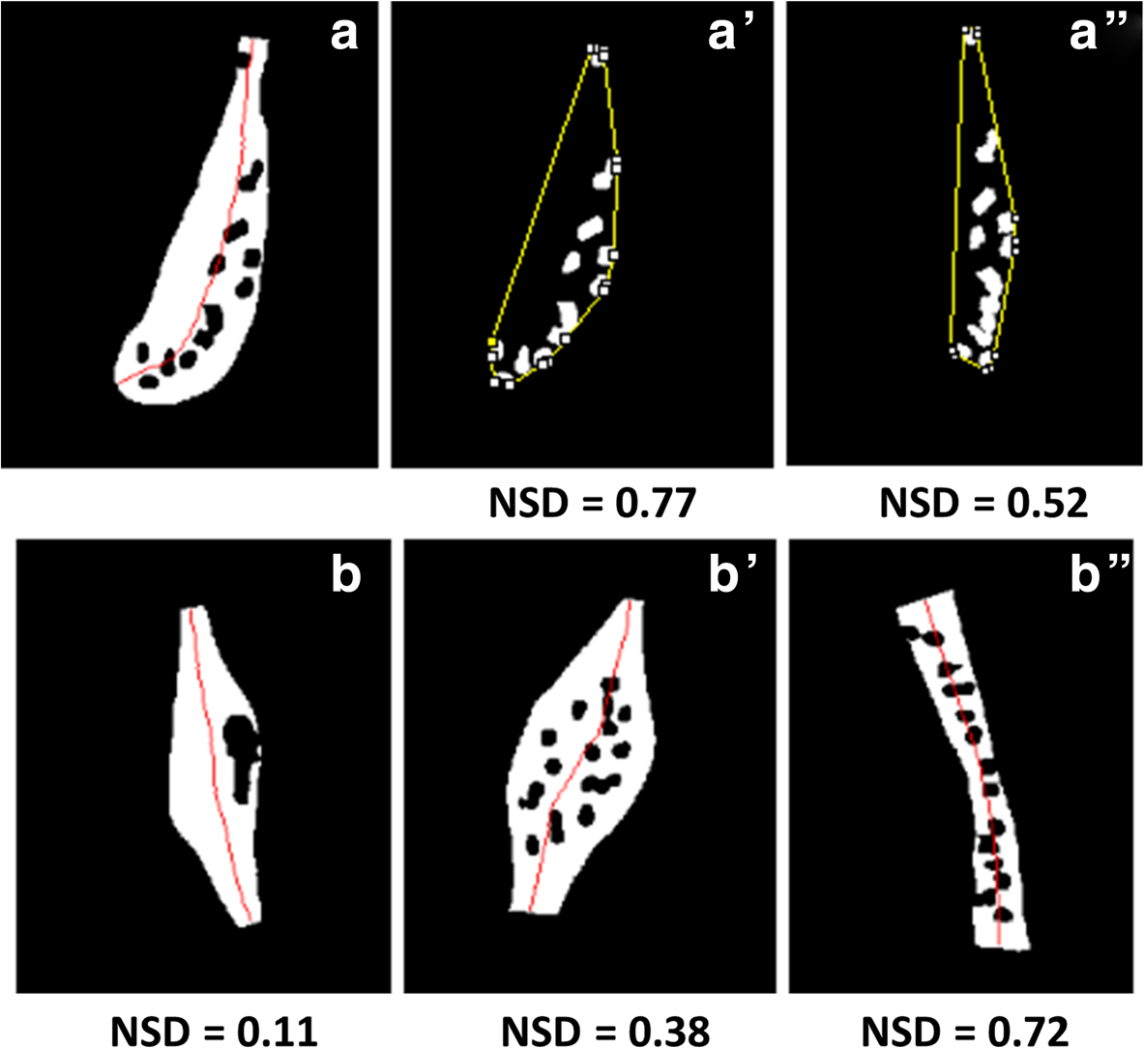
Example showing differences in nuclear spatial density due to change in cell size. (**a**-**a”**) NSD: Nuclear Spatial density. The figure shows straightening of nuclei with respect to the medial axis of nuclei. It compares the nuclear spatial density index for straightened (**a”**) and un-straightened nuclei (**a’**). **b**-**b”** The value of nuclear spatial density is lower for densely packed nuclei (**b**) as compared to evenly distributed nuclei (**b”**)

Aligning the nuclei along the straightened medial axis of muscle cell helps to provide a spatial reference for comparing nuclei from different samples. We assume that the medial axis of muscle cell is straight and adjust the position of nuclei according to the changes in the curvature of the medial axis (Figs. 7 & 8a-a”). The straightening algorithm is as follows:

1. Find the centroid of a nucleus.
2. Find the smallest distance (*dx)* to the centroid from the medial axis of muscle cell. Let the point at the medial axis which has the least distance from the centroid be *m*.
3. Find the length of medial axis (*dy)* between the start of medial axis and point *m*.
4. Find the angle (α), i.e. the angle between the tangent at point *m* of medial axis and y axis.
5. Assuming that the start point of straightened medial axis lies at *x* = X, *y* = 0, then the new coordinate of point *m* is *P(X,dy)* and the centroid of the nucleus is *P(X ± dx, dy)*. X is a constant number such that *X ± dx* is never negative. The sign in *X ± dx* depends on whether the nucleus is located on left or right side of medial axis.
6. Next, Translate the nucleus to point *P(X ± dx, dy)* and rotate the nucleus by an angle α.
7. Repeat steps 1–6 for each nuclei inside the muscle cell.

1. Nuclear spatial density

As mentioned earlier, it has been previously shown that there is an association between nuclear localization and muscle mass change. To confirm this hypothesis, we design a feature that measures the nuclear spatial density with respect to the cell size.

2 $$ \mathrm{Nuclear}\ \mathrm{spatial}\ \mathrm{density}\ \left(\mathrm{NSD}\right)=\frac{\mathrm{Area}\ \mathrm{of}\ \mathrm{convex}\ \mathrm{hull}\ \mathrm{of}\ \mathrm{nuclei}}{\mathrm{Area}\ \mathrm{of}\ \mathrm{muscle}\ \mathrm{cell}} $$

A straightened nuclear structure is used to calculate the convex hull. The convex hull of a nuclear region is the smallest convex set which contains that region [35]. If nuclear spatial density is close to 1, the nuclei are located close to the muscle boundary and are distributed more evenly. Alternatively, a low value of NSD indicates that the nuclei formed a cluster and occupied a small part of the muscle cell. In Fig. 8, the sample *b* has the smallest NSD, resulting from clustering of the nuclei. In sample *b’*, although the nuclei are more spread out, the NSD is not as high as in sample *b”*. This is because sample *b’* has a larger cell size than sample *b”*.

2) Longitudinal nuclear spread

**Fig. 9:**
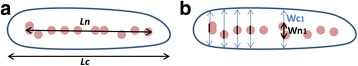
Schematic diagram explaining the derivation of nuclear pattern features. **a** Normalized longitudinal nuclear spread. **b** Normalized lateral nuclear spread

Longitudinal nuclear spread characterizes the polar and anti-polar migration of nuclei. It is defined as the distance between the extremes of the nuclei along the medial axis and denoted as *L*_*n*_. The normalized migration of nuclei along the medial axis/normalized longitudinal nuclear spread *NM*_*lon*_ is defined as:

3 $$ {NM}_{lon}={L}_n/{L}_c $$

where *L*_*n*_ is the longitudinal nuclear spread and *L*_*c*_ is the length of the muscle cell (Fig. 9a). A high *NM*_*lon*_ indicates that the nuclei are close to the poles of the muscle cell and a low value indicates that they are far.

3) Lateral nuclear spread

Lateral nuclear spread characterizes the movement of nuclei away from the medial axis of muscle during late stages of pupal development. The lateral nuclear spread *M*_*lat*_ is defined as:

4 $$ {M}_{lat}=\frac{1}{u}{\sum}_{i=1}^{i= u} W{n}_i $$

where $W{n}_i$ is the width of the nuclear structure at the *i* ^*th*^ location on the medial axis, and *u* is the number of samples taken along medial axis (Fig. 9b). All of the samples are collected at equal interval along medial axis. A high *M*_*lat*_ indicates an increase in the distance between nuclei along the width of muscle i.e. two-row formation of nuclei.

**Fig. 10:**
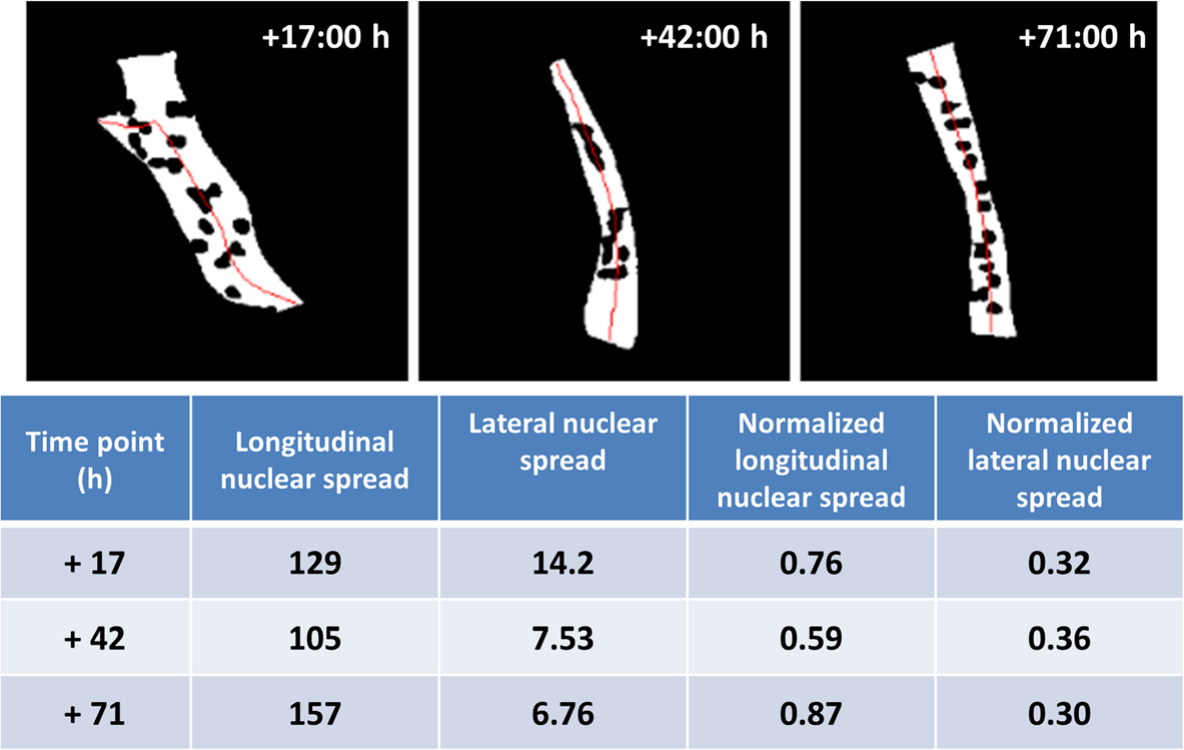
Comparison of nuclear pattern features of a muscle at three different time points. The muscle shown in the figure is from a control samples. At +71 h, the muscle has highest *M*_*lon*_ and *NM*_*lon*_ because the nuclei are closer to the poles of the muscle cell than at other time points. Whereas, the muscle has highest *M*_*lat*_ at +17 h; because the nuclei are more spread out along the width of muscle. The value of *NM*_*lat*_ is approximately similar at three time points. This indicates that at these three time points, the spread of nuclei with respect to width of muscle is similar

In order to quantify the influence of muscle mass change on lateral displacement of nuclei, we also designed normalized lateral nuclear spread. Normalized lateral nuclear spread *NM*_*lat*_ is defined as:

5 $$ {NM}_{lat}=\frac{1}{u}{\sum}_{i=1}^{i= u} W{n}_i/ W{c}_i $$

where $W{c}_i$ is the width of muscle cell at the *i* ^*th*^ location on the medial axis. A high *NM*_*lat*_ indicates that the nuclei are close to the boundary of the muscle cell. A few examples of the values of longitudinal nuclear spread and lateral nuclear spread are shown in Fig. 10.

### Implementation of algorithm

The nuclear pattern analysis algorithms were implemented in Java and incorporated as a part of the FMAj tool. We have used two external libraries: Mexican hat filter [27] and hull and circle plugin [35]. The nuclear analysis module in FMAj is divided into three sections: nuclear segmentation, nuclear classification and nuclear feature generation. Nuclear segmentation is performed on complete image. Whereas, nuclear classification is performed on nuclei inside muscles using muscle boundaries. The nuclear features generated by FMAj are stored in a MySQL database. The analysis of the features was done in excel and FMAj.

## Results

### Evaluation of nuclear segmentation

We evaluated the performance of segmentation at object level rather than pixel level. We manually counted the false negative, false positive and true positive by comparing the segmentation results with original image. False positive (FP) is the count of segmented objects which were not nuclei. True positive (TP) is the count of segmented objects which were nuclei. False negative (FN) is the count of nuclei which were not segmented. We evaluated the segmentation results for 140 time points that were selected from two *Atg9* samples. We used two parameters for evaluation:

$$ \mathrm{False}\ \mathrm{negative}\ \mathrm{rate}=\mathrm{FN}/\left(\mathrm{FN}+\mathrm{TP}\right) $$

6 $$ \mathrm{False}\ \mathrm{positive}\ \mathrm{rate}=\mathrm{FP}/\left(\mathrm{FP}+\mathrm{TP}\right) $$

The false negative rate is the ratio of false negative and the total number of actual nuclei; whereas, the false positive rate is the ratio of false positive and the total number of nuclei segmented.

The performance of thresholding segmentation with LoG filtering is compared with thresholding (Otsu) without LoG filtering in Table 1. As shown in Table 1, LoG based method has zero false negative rate and 0.024 false positive rate. It means that LoG based method correctly segments every nuclei; however it also segments some non-nuclei high intensity spots (imaging noise). In comparison, Otsu thresholding without LoG filtering has false negative rate of 0.11 which is not suitable for spatial pattern analysis. The results of LoG based segmentation without bi-exponential smoothening is also shown in Table 1. The false positive rate is higher in the case of segmentation without smoothening as compared to with smoothening. This verifies that smoothening of our images is necessary to remove imaging noise.

### Evaluation of nuclear classification

λ = 7 is used for nuclei classification in our dataset. In order to find the correct λ for our dataset, we used a training dataset to calculate the performance of classification for different thresholds. We calculated following parameters for classification performance evaluation: accuracy, false positive rate and false negative rate.

7 $$ \mathrm{Accuracy}=\left(\mathrm{TP}+\mathrm{TN}\right)/\left(\mathrm{TP}+\mathrm{FP}+\mathrm{TN}+\mathrm{FN}\right) $$

**Fig. 11:**
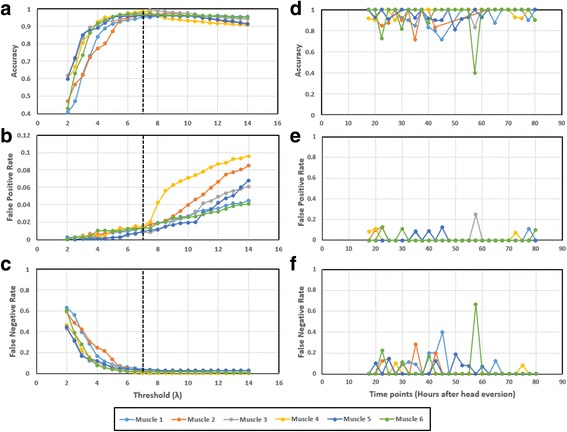
Performance evaluation of nuclear classification at different threshold values and different development stages. **a**-**c** The graphs compare the performance of nuclear classification at different thresholds using following parameters i.e. accuracy (**a**), false positive rate (**b**) and false negative rate (**c**). Each series in the graph represents a different muscle cell. The *black dotted line* indicate the threshold value at which accuracy is highest and error rates are lowest. This threshold value is used for nuclear classification. **d**-**f** The graphs compare the performance of nuclear classification at different stages of pupal development using following parameters i.e. accuracy (**d**), false positive rate (**e**) and false negative rate (**f**). Large movements of internal nuclei results in low accuracy and high error during between +20 and +50 h. In comparison, accuracy is high after +50 h due to slow movement of internal nuclei

We measured the false negative, false positive, true negative and true positive by comparing the classification results with a ground truth which was generated manually using FMAj [23]. False positive (FP) is the count of external nuclei which were falsely classified as internal nuclei. True positive (TP) is the count of correctly classified internal nuclei. False negative (FN) is the count of internal nuclei falsely classified as external nuclei. True negative (TN) is the count of correctly classified external nuclei. The performance evaluation was done for 6 different muscle cells from different genotypes i.e. two samples from control, *Atg9* and *Atg12*; data was generated from at least 50 time points per muscle cell. We visualized accuracy, false positive rate and false negative rate for these 6 muscles in a graph as shown in Fig. 11a-c. The threshold at which accuracy was highest and error rates were minimum was selected for classification of nuclei (Shown by black dotted line) i.e. λ = 7.

Thus, we measured the performance of our nuclear classification algorithm at λ = 7, for the same 6 muscles which were used for calculation of λ. The results are as follows: average accuracy = 96.9 ± 1.2%, average false positive rate = 1.2 ± 0.2%, average false negative rate = 2.1 ± 1.3%. A comparison of nuclear classification performance evaluation during different stages of pupal development is shown in Fig. 11d-e. It can be observed that the accuracy of nuclear classification suffers due to the large movements of internal nuclei between 20 h and 50 h after head eversion. Whereas, during later stages of pupal development, the reduction in movement of internal nuclei results in high accuracy and low false positive and negative rates.

### Results of Myonuclear spatial pattern analysis

We previously described the effects of genetic perturbations on nuclear migration remodeled muscles [14]. Here, we used the nuclear spatial pattern analysis algorithm to quantify nuclear distribution in *Cp1* ^shRNA^, *Atgs* ^shRNA^ and control muscles. To compare different genotypes, we calculated the nuclear features for each genotype and performed a non-parametric Mann-Whitney U test. The significance test was performed for nuclear features at every time point. We plotted −1*log_10_(P-val) for each time point where nuclear features had been calculated. Value of −1*log_10_(P-val) above 1.3 (P-val = 0.05) is considered significant. We used head eversion (HE) as a temporal reference to compare different samples. Time was represented as hours (h) after head eversion. DIOM muscle from 3rd segment of pupa abdomen was used for this analysis. We have also compared the properties of nuclear tracks between different genotypes like start and end time of track, length of track, nucleus speed etc. (Additional file 1: Table S2). It shows that external nuclei display high speed and large movements compared to internal nuclei irrespective of genotype.

### *Cp1* participates in the anti-polar/polar migration of nuclei

**Fig. 12:**
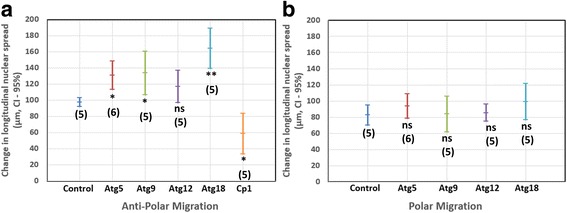
Comparison of change in longitudinal nuclear spread during polar and anti-polar migration between different genotypes. The graph shows the mean of difference in the longitudinal nuclear spread *L*_*n*_ between different genotype for anti-polar migration (**a**) and polar migration (**b**). The error bars show the 95% confidence interval. During anti-polar migration, the movement of nuclei towards the muscle centre is higher in *Atg5*, *Atg9* and *Atg18* compared to control. Therefore, at the end of anti-polar migration the distance between extreme nuclei and poles is significantly large in above mentioned *Atg* genes. However, during polar migration, the total distance moved by nuclei towards poles in *Atgs* is not significantly different from control. This would indicate that the reduced expanse of nuclei in *Atgs* compared to control during polar migration (Fig. 14) is due to positioning of nuclei at large distances from poles at beginning of polar migration. The reduced anti-polar migration in *Cp1* is also shown by the small change in expanse of nuclear structure as compared to control. *P < 0.05, **P < 0.01, ***P < 0.001, ns  =  not significant (*P* > 0.05)

**Fig. 13:**
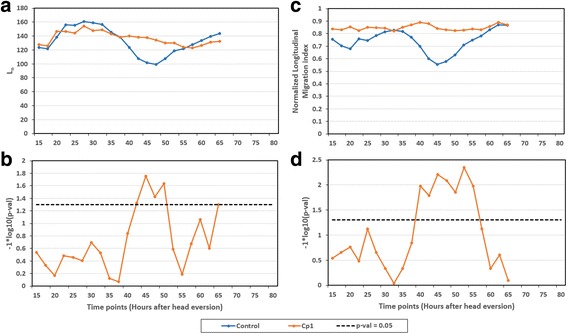
Longitudinal nuclear spread quantifies the effect of *Cp1* knockdown on anti-polar migration. **a** & **c** Graphical comparisons of *L*_*n*_ (Distance between the extremes of the nuclei along the medial axis) and normalized longitudinal nuclear spread *NM*_*lon*_ between control and *Cp1* mutant. Due to absence of anti-polar movement of muscles around +50 h in *Cp1* mutants; the value of both *L*_*n*_ and *NM*_*lon*_ is high as compared to control. **b** & **d** Significance graph. For each population, statistics were derived from 5 to 7 DIOM1. The muscles were from segment 3 of abdomen. The graph on top show average values of the features. The horizontal dotted lines in the significance graphs represent the *p*-value 0.05

Quantitative nuclear pattern analysis confirmed that the knockdown of *Cp1* affected anti-polar nuclear migration in early pupation (Additional file 1: Figure S1c, d). In control muscles, anti-polar migration reduced the expanse of nuclei along the medial axis (*L*_*n*_) by ~97.5 μm between +28.9 h (205.8 μm) and +46.6 h (108.4 μm) (Additional file 1: Table S3, Fig. 1a, b). By contrast, *Cp1* silencing resulted in reduction of *L*_*n*_ by ~58.6 μm between +23.5 h (209.8 μm) and +54.1 h (151.3 μm) (Fig. 12a). Unlike control, where nuclear polar migration increased the value of *L*_*n*_ by ~82.6 μm between +46.6 (108.4 μm) h and +72.1 h (190.9 μm); *Cp1* RNAi did not show polar migration. Due to decreased anti-polar migration in *Cp1* RNAi, the values of *L*_*n*_ are significantly different for control and *Cp1* RNAi between +42 h and +52 h (Fig. 13a, b).

The normalized longitudinal nuclear spread (*NM*_*lon*_), indicated that the reduction in expanse of nuclei along medial axis of muscle (*L*_*n*_) in *Cp1* RNAi is due to shortening of muscle cells. This phenomenon was confirmed by very small change in values of *NM*_*lon*_ for *Cp1* RNAi between +32.5 h and +60 h (Fig. 13c). Between +32.5 h and +45 h, *NM*_*lon*_ values changed only by 0.2 (0.82 ± 0.07–0.84 ± 0.05) for *Cp1* RNAi as compared to 0.27 (0.83 ± 0.05–0.55 ± 0.13) for control. Similarly, between +45 h and +60 h, *NM*_*lon*_ values changed only by 0.2 (0.84 ± 0.05–0.85 ± 0.02) for *Cp1* RNAi as compared to 0.28 (0.55 ± 0.13–0.83 ± 0.03) for control. The significantly high values of *NM*_*lon*_ for *Cp1* RNAi as compared to control between +40 h and +57 h indicate that the knockdown of *Cp1* resulted in absence of anti-polar migration (Fig. 13d).

### Silencing of *Atg9* and *Atg18* increases the anti-polar migration and decreases the polar migration of nuclei

**Fig. 14:**
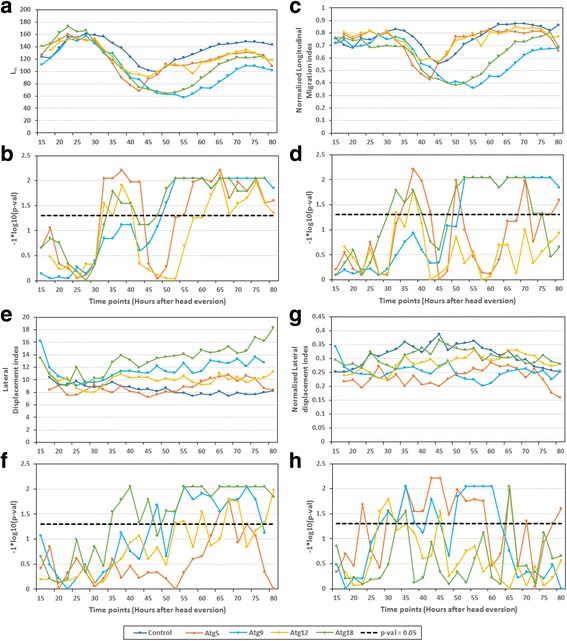
Nuclear pattern features quantifies the effect of loss of autophagy on nuclear distribution. The figure compares following nuclear pattern features y in muscles expressing *Atg5* RNAi, *Atg9* RNAi, *Atg12* RNAi, *Atg18* RNAi and control: (**a**) *L*_*n*_. (**c**) Normalized longitudinal nuclear spread. **e** Lateral nuclear spread. **g** Normalized lateral nuclear spread. The graphs show average values of the features. **b**, **d**, **f**, **h** Significance graph. For each population, statistics were derived from 5 DIOM1 (Segment 3). The horizontal dotted lines in the significance graphs represent the *p*-value 0.05

RNAi of *Atg5*, *Atg9* and *Atg18* affected myonuclear migration. While knockdown of all *Atgs* RNAi did not block anti-polar and polar nuclear migration (Additional file 1: Figure S2), the silencing of *Atg9* and *Atg18* resulted in prolonged anti-polar migration of nuclei (7.5 h (median) delay for *Atg9* RNAi and 4 h (median) delay for *Atg18*) and larger anti-polar movement of nuclei (between +27.4 h and +53 h for *Atg9* and between +22.4 h and +48.1 h for *Atg18*) as compared to control (between +28.9 h and +46.6 h) (Fig. 14a, b). The reduction in value of *L*_*n*_ during anti-polar migration in *Atg5*, *Atg9, Atg18* and control was ~130.8 μm, ~133.9 μm, ~164.4 μm and ~97.5 μm; this indicates that nuclei are farther from poles in *Atg5*, *Atg9* and *Atg18* as compared to control (Fig. 12a, Additional file 1: Table S3). Knockdown of *Atg9* and *Atg18* also reduced the polar migration of nuclei (Between +55 h and +72.5 h for *Atg9* and between +50 h and +72.5 h for *Atg18*), resulting in larger distances between pole and nearest nucleus (Fig. 14a, b). The value of *L*_*n*_ at the end of polar migration in *Atg9, Atg18* and control was ~141.6 μm, ~162.4 μm and ~190.9 μm approximately; this indicates reduced polar migration in *Atg9* and *Atg18* (Additional file 1: Table S3). However, it is important to note that in spite of lower longitudinal nuclear spread in *Atgs*, there is no significant difference between the changes in longitudinal nuclear spread (between start and end of polar migration) in *Atgs* and control as shown in Fig. 12b.

The significant difference in *NM*_*lon*_ between the control and *Atg9* and *Atg18* RNAi after +50 h and +46 h respectively proved that the polar/anti-polar migration of nuclei in *Atg9* RNAi and *Atg18* RNAi is not affected by muscle contraction/elongation (Fig. 14c, d). The effects of silencing *Atg5* and *Atg12* on nuclear migration are not as prominent as *Atg9* and *Atg18* silencing. In both *Atg5* and *Atg12*, the values of *L*_*n*_ is significantly less than control at the end of polar migration (~169 μm at +72.1 h for *Atg5*, ~177.1 μm at +72.2 h for *Atg12* and ~190.9 μm for control), although not as low as *Atg9* and *Atg18*; this indicates reduced polar migration (Additional file 1: Table S3). Also, there was no significant difference in the values of *NM*_*lon*_ in *Atg5* and *Atg12* RNAi during the polar migration. These findings indicate that the decreased polar migration caused by silencing of *Atg5* and *Atg12* could be due to contraction of muscle cells.

### Lateral myonuclear displacement Atg knockdowns correlates with increased diameter of muscles

As previously reported [14], loss of autophagy resulted in nuclei being arranged in two rows in late metamorphosis as compared to single row formation in control muscles. We used lateral nuclear spread (*M*_*lat*_) and normalized lateral nuclear spread (*NM*_*lat*_) to quantify the lateral movement of nuclei perpendicular to the medial axis of muscles. In *Atg9* and *Atg18* RNAi, the value of *M*_*lat*_ was significantly higher in controls after +53 h and +50 h, respectively; suggesting that nuclei moved away from the muscle’s medial axis and arranged in a two-row formation (Fig. 14e, f). Interestingly, we did not observe a similar trend in *NM*_*lat*_ for *Atg9* and *Atg18* RNAi (Fig. 14g). We found the values of *NM*_*lat*_ were significantly lower for *Atg9* RNAi compared to controls between +50 h and +70 h (Fig. 14h). This indicates that between +50 h to +70 h, the nuclei were located away from the muscle cell boundary as compared to control, even though they were arranged in two rows. However, after +70 h, values of *NM*_*lat*_ were not significantly different between control and *Atg9* RNAi. Whereas, in *Atg18* RNAi, the value of *NM*_*lat*_ were not significantly different from control throughout pupal development. Therefore, the lateral displacement of nuclei might be dependent on the change in muscle width in *Atg9* and *Atg18* RNAi. The increase in width of muscle cell due to loss of autophagy could be increasing the distance between nuclei resulting in two-row formation. In case of *Atg5* and *Atg12*, the lateral displacement features were unable to statistically prove the two-row formation, despite being confirmed visually, indicating a lower penetration of the phenotype.

### The anti-polar/polar migration and lateral displacement of nuclei affect the spatial density of nuclei in muscles

**Fig. 15:**
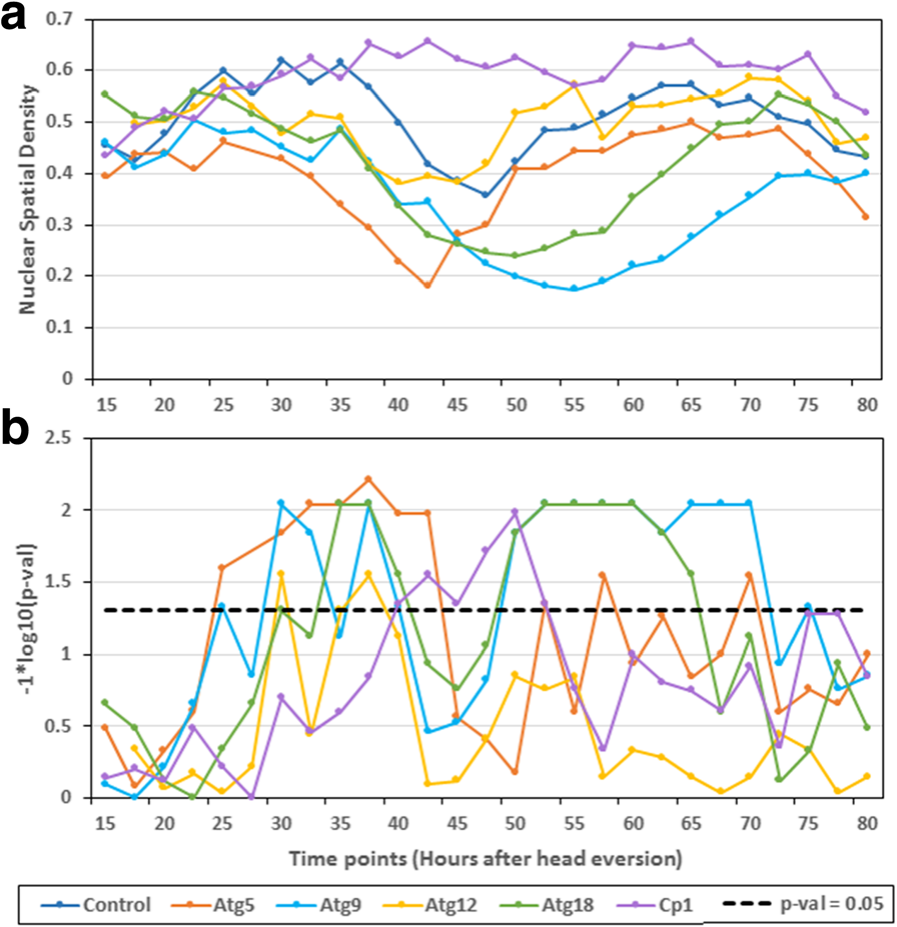
Nuclear spatial density index quantifies the nuclear distribution with respect to the muscle mass change. **a** The figure compares average nuclear spatial density index of nuclei in muscles expressing *Cp1* RNAi, *Atg5* RNAi, *Atg9* RNAi, *Atg12* RNAi, *Atg18* RNAi and control. **b** Significance graph. Statistics were derived from the same DIOM1 used in previous two figures. The *horizontal dotted lines* in the significance graphs (*bottom panels*) represent the *p*-value 0.05

During early stages of pupal development, differences in spatial density of nuclei were observed between Atgs (*Atg5*, *Atg9* & *Atg18*) and control. In *Atg5* RNAi, significant reduction in NSD was observed between +25 h to +42.5 h compared to control. This observation could be the result of larger anti-polar migration and smaller lateral displacement of nuclei with respect to width of muscle. Significant reduction in spatial density of nuclei was also observed in *Atg9* (between +30 h to +40 h) and *Atg18* (between +35 h to +40 h)*.* We also observed that the spatial density of nuclei with respect to the muscle area in *Atg9* and *Atg18* was significantly lower than the control between +50 h to +70 h and +50 h to +65 h respectively (Fig. 15a, b). It indicates that the knockdown of both *Atg9* and *Atg18* increased the area devoid of nuclei in muscles. Since, the lateral displacement of nuclei increases between +50 h to +70 h in *Atg9* and *Atg18* RNAi; the reduced polar migration could be the reason for low spatial density of nuclei (NSD) with respect to the muscle area. As opposed to other *Atgs*, *Atg12* knockdown did not affect the spatial density of nuclei with respect to the muscle area despite the two-row formation. Increased polar migration during later pupal development could be one of the reasons behind the control like nuclear spatial density in *Atg5* and *Atg12* RNAi as compared to *Atg9 and Atg18* (Fig. 14a, b).

## Discussion

We previously found that muscle atrophy in remodelled muscles is accompanied by extensive myonuclear migration [14]. To better understand the process of nuclear migration in muscle cells, we designed an algorithm to extract a set of nuclear spatial pattern features. Apart from the semi-automated muscle segmentation, all the processes including nuclear segmentation and tracking are performed in a fully automated fashion, thus enabling a more reproducible analysis of sizeable time-series image data. Nuclear classification results have been improved by introducing a tracking based algorithm which exploits the differences in motion of external and internal nuclei to classify them. Also, the adjustment of position of nuclei based on muscle cell displacement reduced the classification errors caused by large movements of the muscle cell. In some cases, abrupt change in shape of muscle does affect the nuclear tracking result. If the shape change is only between two time points, our algorithm handles this case by creating a new track for the affected nuclei. Therefore, the results of classification are not affected. However, if muscle keeps changing shape for larger duration of time, the performance of classification algorithm will decrease. In our dataset, such cases are very few. In the future, further work can be done to improve the methodology of nuclei adjustment based on displacement of muscle cell centroid. In order to reduce the effect of muscle cell shape change in adjustment of nuclei, a shape matching using criteria like chamfer distance could be used to align muscle cells.

Multiple nuclear spatial pattern features have been designed, each catering to a specific type of nuclear distribution. The nuclear spatial density index measures how densely the nuclei are packed; however, they cannot quantify the localization of the nuclei in the muscle cell. To accomplish this, we have designed new features that can quantify the distribution of nuclei along the medial axis and along the width of the muscle cell, termed longitudinal nuclear spread and lateral nuclear spread respectively.

In this study, the abnormal nuclei arrangement in *Cp1* and *Atgs* RNAi has been analyzed statistically using nuclear spatial pattern features. According to the myonuclear domain theory, nuclei should be evenly distributed in healthy muscle [36], which is a phenotype we observed in *Cp1* mutant; whereas central positioning of nuclei, which has been associated with Central nuclear myopathies, was observed in control and *Atgs* RNAi during the mid-pupal stage. Knockdown of *Atg9* and *Atg18* RNAi resulted in more densely packed (longitudinally) nuclei as compared to control. This indicates that *Atg9* and *Atg18* help in the central positioning of nuclei by regulating their anti-polar migration. We also observed that the effect of silencing of genes on nuclear migration is more prominent in *Atg9* and *Atg18* as compared to *Atg5* and *Atg12*. *Atgs* were also involved in positioning of nuclei along the width of muscle. In order to understand how these genes alter the nuclear spatial pattern, further analysis is required. However, it is clear that the metamorphosis in *Drosophila* provides a good platform to study nuclear migration and localization in muscles.

## Conclusion

Understanding the association between the dynamics of myonuclear localization and change in muscle mass requires extensive quantitative analysis of a large number time series images collected in vivo. We achieved it by combining time lapse in vivo imaging of *Drosophila* metamorphosis with a semi-automated nuclear pattern analysis algorithm. We developed new nuclear features to characterize the dynamics of nuclear distribution in time-lapse images of *Drosophila* metamorphosis. Image quantification improved our understanding of phenotypic abnormalities in nuclear distribution resulting from gene perturbations. Further analysis on larger number of genes is required to understand in depth the molecular mechanisms behind the myonuclear localization patterns. Therefore in vivo imaging and quantitative image analysis of *Drosophila* metamorphosis promise to provide novel insights into the relationship between muscle wasting and myonuclear positioning.

## Additional file

Additional file 1: Sample table of nuclear tracks, results of anti-polar/polar migration analysis and figures showing myonuclear distribution phenotypes observed in previous study. (DOCX 5608 kb)

## Abbreviations

AHE: After head eversion
DIOM: Dorsal internal oblique muscle
FMAj: Fly muscle analysis in java
GFP: Green fluorescent protein
LoG: Laplacian of Gaussian
MHC: Myosin heavy chain
MIP: Maximum intensity projection
mKO: Monomeric Kusabira Orange
MND: Myonuclear domain
NSD: Nuclear spatial density
RNAi: RNA interference
TLM: Time-lapse microscopy
