# Supplementary material for: Spatial pattern analysis of nuclear migration in remodelled muscles during Drosophila metamorphosis
Source: BMC Bioinformatics. 2017 Jul 10;18:329. doi: 10.1186/s12859-017-1739-0 (PMC5504860; doi:10.1186/s12859-017-1739-0)

**Additional files**

**Table S1. Representation of nuclear tracks in tabular manner**

Each column of the table is one track. The column contains the unique label of the nuclei which are part of the track. Zero value indicate that the track has terminated. The tracks shown here correspond to the muscle shown in Figure 6. Tracks shown in red were classified as external nuclei and their contours were removed from segmentation results. aHE: after Head Eversion

| **Time, aHE [Hours]** | **Time Frame Number** | | **Tracks** | | | | | | | | | | | | | | | | | |
| --- | --- | --- | --- | --- | --- | --- | --- | --- | --- | --- | --- | --- | --- | --- | --- | --- | --- | --- | --- | --- |
|  |  |  | **Track 1** | **Track 2** | **Track 3** | **Track 4** | **Track 5** | **Track 6** | **Track 7** | **Track 8** | **Track 9** | **Track 10** | **Track 11** | **Track 12** | **Track 13** | **Track 14** | **Track 15** | **Track 16** | **Track 17** | **Track 18** |
| 36 | | 87 | 100 | 101 | 102 | 103 | 104 | 105 | 106 | 107 | 108 | 109 | 110 | 111 |  |  |  |  |  |  |
| 36.5 | | 88 | 0 | 100 | 103 | 104 | 105 | 106 | 107 | 108 | 110 | 111 | 112 | 113 | 101 | 102 | 109 |  |  |  |
| 37 | | 89 | 0 | 100 | 102 | 103 | 104 | 105 | 0 | 106 | 107 | 108 | 109 | 110 | 101 | 0 | 111 |  |  |  |
| 37.5 | | 90 | 0 | 100 | 101 | 102 | 103 | 104 | 0 | 107 | 109 | 108 | 110 | 111 | 0 | 0 | 112 | 105 | 106 |  |
| 38 | | 91 | 0 | 100 | 102 | 103 | 104 | 105 | 0 | 106 | 108 | 107 | 109 | 110 | 0 | 0 | 0 | 0 | 0 | 101 |

**Table S2. Representative nuclear tracks. External nuclei corresponding to fragmented dead muscles move faster than nuclei tracked in remodelled muscles.**

The data in the table was generated using the nuclear tracks created during nuclear spatial pattern analysis. Rows in green and red show the internal and external nuclei respectively. aHE: after Head Eversion

| **Nuclear Tracks Table** | | | | | | | | | |
| --- | --- | --- | --- | --- | --- | --- | --- | --- | --- |
| **Track ID** | **Genotype** | **Location of the nucleus** | **Start Time aHE [min]** | **End Time aHE [min]** | **Track length [Number of frames]** | **Track length [min]** | **Distance [microns]** | **Average speed [microns/hour]** |  |
| 2 | Atg9-RNAi | Intracellular | 1230 | 1740 | 17 | 510 | 94.24 | 11.08706 |  |
| 13 | Atg9-RNAi | Intracellular | 1230 | 1890 | 22 | 660 | 137.64 | 12.51273 |  |
| 207 | Atg9-RNAi | Intracellular | 3630 | 5130 | 50 | 1500 | 207.08 | 8.2832 |  |
| 208 | Atg9-RNAi | Intracellular | 3660 | 5100 | 49 | 1440 | 147.56 | 6.022857 |  |
| 76 | Atg9-RNAi | Extracellular | 1740 | 1890 | 5 | 150 | 99.2 | 39.68 |  |
| 4 | Atg9-RNAi | Extracellular | 1230 | 1530 | 10 | 300 | 104.16 | 20.832 |  |
| 1 | Cp1-RNAi | Intracellular | 1050 | 3810 | 92 | 2760 | 398.04 | 8.653043 |  |
| 30 | Cp1-RNAi | Intracellular | 1320 | 2040 | 24 | 720 | 128.96 | 10.74667 |  |
| 94 | Cp1-RNAi | Intracellular | 2040 | 3810 | 59 | 1770 | 226.92 | 7.692203 |  |
| 143 | Cp1-RNAi | Extracellular | 3390 | 3540 | 5 | 150 | 54.56 | 21.824 |  |
| 3 | Chro-RNAi | Intracellular | 840 | 1950 | 37 | 1110 | 198.4 | 10.72432 |  |
| 45 | Chro-RNAi | Intracellular | 1230 | 2070 | 28 | 840 | 162.44 | 11.60286 |  |
| 215 | Chro-RNAi | Intracellular | 3330 | 4890 | 52 | 1560 | 189.72 | 7.296923 |  |
| 21 | Chro-RNAi | Extracellular | 930 | 1110 | 6 | 180 | 85.56 | 28.52 |  |

**Table S3. Comparison of polar and anti-polar myonuclear migration between different genotypes**

Features (mean ±std) were calculated using nuclear spatial pattern analysis. ‘Total Distance’ column lists the difference in the expanse of nuclear structure (*L_n_*) between start and end of migration. n=Number of muscle cells. Last column lists the p-values obtained by comparing ‘Total Distance’ parameter between control and other genotypes. Rows in green show the genotypes with significant p-values.

1. Anti-polar migration

| **RNAi** | **Start of anti-polar migration [Hour aHE]** | **L_n_ at start of Anti-polar migration [µm]** | **End of Anti-polar migration [Hour aHE]** | **L_n_ at end of Anti-polar migration [µm]** | **Total Distance [µm]** | **n** | **P-value** |
| --- | --- | --- | --- | --- | --- | --- | --- |
| **Control** | 28.9±2.3 | 205.8±18.2 | 46.6±3.7 | 108.4±22.7 | 97.5±6.2 | 5 | - |
| ***Cp1*** | 23.9±2.5 | 203.6±25.5 | 53.1±4.8 | 147.6±11 | 56±22.8 | 5 | 0.021 |
| ***Atg5*** | 23±3 | 206.3±11.8 | 41.9±1.1 | 75.4±17.9 | 130.8±22.1 | 6 | 0.044 |
| ***Atg9*** | 27.4±1.5 | 191.7±26.9 | 53±7.6 | 57.7±13.2 | 133.9±30.6 | 5 | 0.021 |
| ***Atg12*** | 23.4±3.2 | 208.5±13.5 | 42.3±2.2 | 91.5±18.1 | 117.1±22.7 | 5 | 0.174 |
| ***Atg18*** | 22.4±1.4 | 227.6±36.7 | 48.1±3.5 | 63.24±27.4 | 164.4±28.5 | 5 | 0.009 |

1. Polar migration

| **RNAi** | **Start of polar migration [Hour aHE]** | **L_n_ at start of polar migration [µm]** | **End of polar migration [Hour aHE]** | **L_n_ at end of polar migration [µm]** | **Total Distance [µm]** | **n** | **P-value** |
| --- | --- | --- | --- | --- | --- | --- | --- |
| **Control** | 46.6±3.7 | 108.4±22.7 | 72.1±2.6 | 190.9±12.5 | 82.6±13.9 | 5 | - |
| ***Cp1*** | - | - | - | - | - |  | - |
| ***Atg5*** | 41.9±1.1 | 75.4±17.9 | 72.1±1.1 | 169±8.9 | 93.6±19 | 6 | 0.273 |
| ***Atg9*** | 53±7.6 | 57.7±13.2 | 73.9±3.6 | 141.6±14.9 | 83.8±25 | 5 | 0.916 |
| ***Atg12*** | 42.3±2.2 | 91.5±18.1 | 72.2±0.8 | 177.1±12.5 | 85.6±12.3 | 5 | 1.0 |
| ***Atg18*** | 48.1±3.5 | 63.24±27.4 | 72.8±3.7 | 162.4±11.9 | 99.2±25.6 | 5 | 0.250 |

###### **Figure S1. Silencing of *Cp1* affect the distribution of nuclei along the medial axis in persistent muscles**

(a) & (b) In control, the nuclei move in anti-polar direction at +45 h (White arrow) followed by polar migration. (c) & (d) In *Cp1* RNAi, the anti-polar migration of nuclei is absent. At + 45 h, the nuclei located at the poles is shown by white arrowheads.


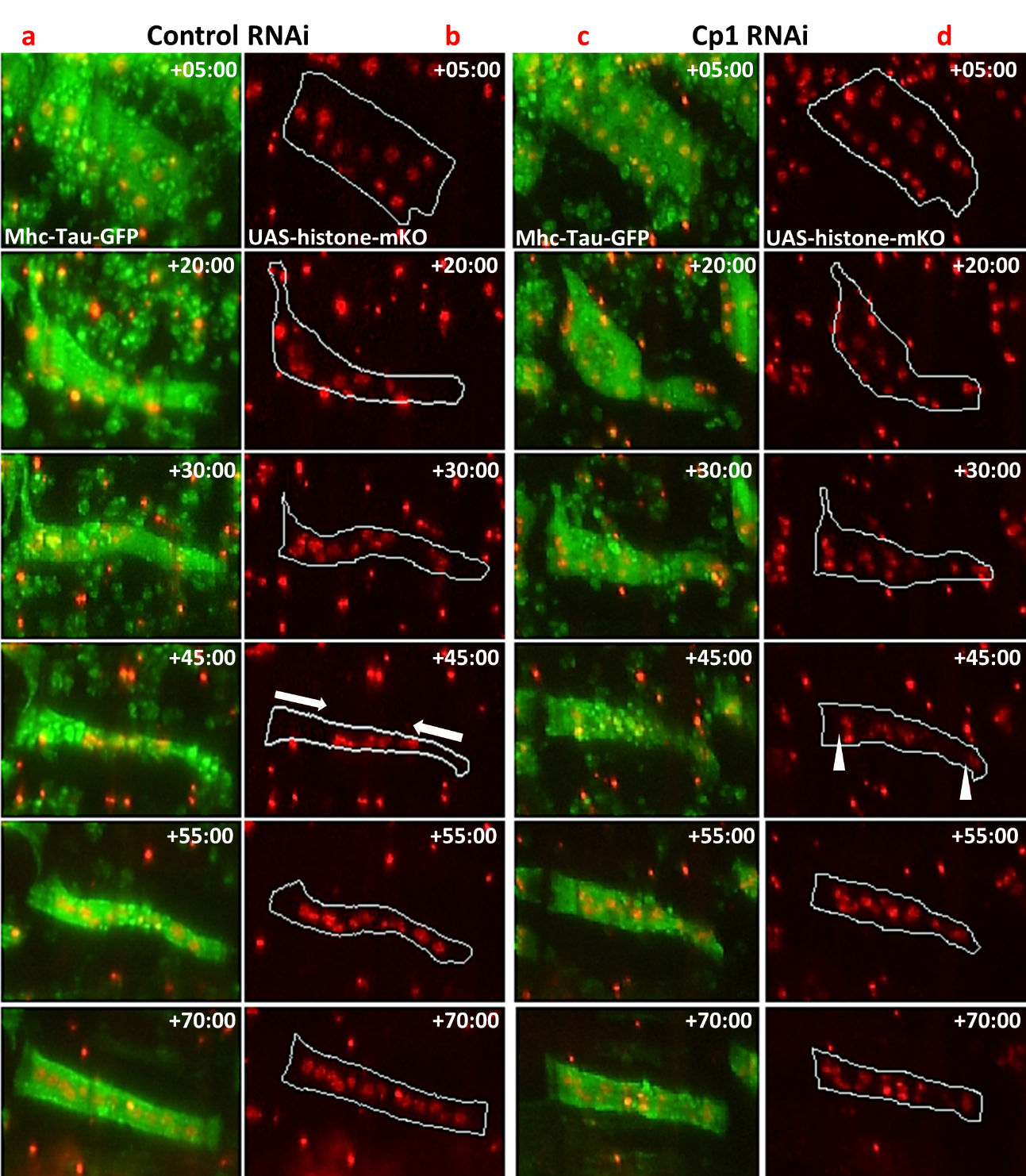


###### **Figure S2. Silencing of *Atgs* affect both lateral and longitudinal migration of nuclei in persistent muscles**

###### The figure shows the nuclei distribution in control (a) and in muscles expressing *Atg18* RNAi (b), *Atg9* RNAi (c), *Atg12* RNAi (d) and *Atg5* RNAi (e). The yellow arrowhead shows the centrally clustered nuclei in *Atg9* and *Atg18* at + 50 h. During late pupal development, the nuclei in control form one row. Whereas in all *Atg* genes, the nuclei form two rows (Shown by a cyan arrowhead).


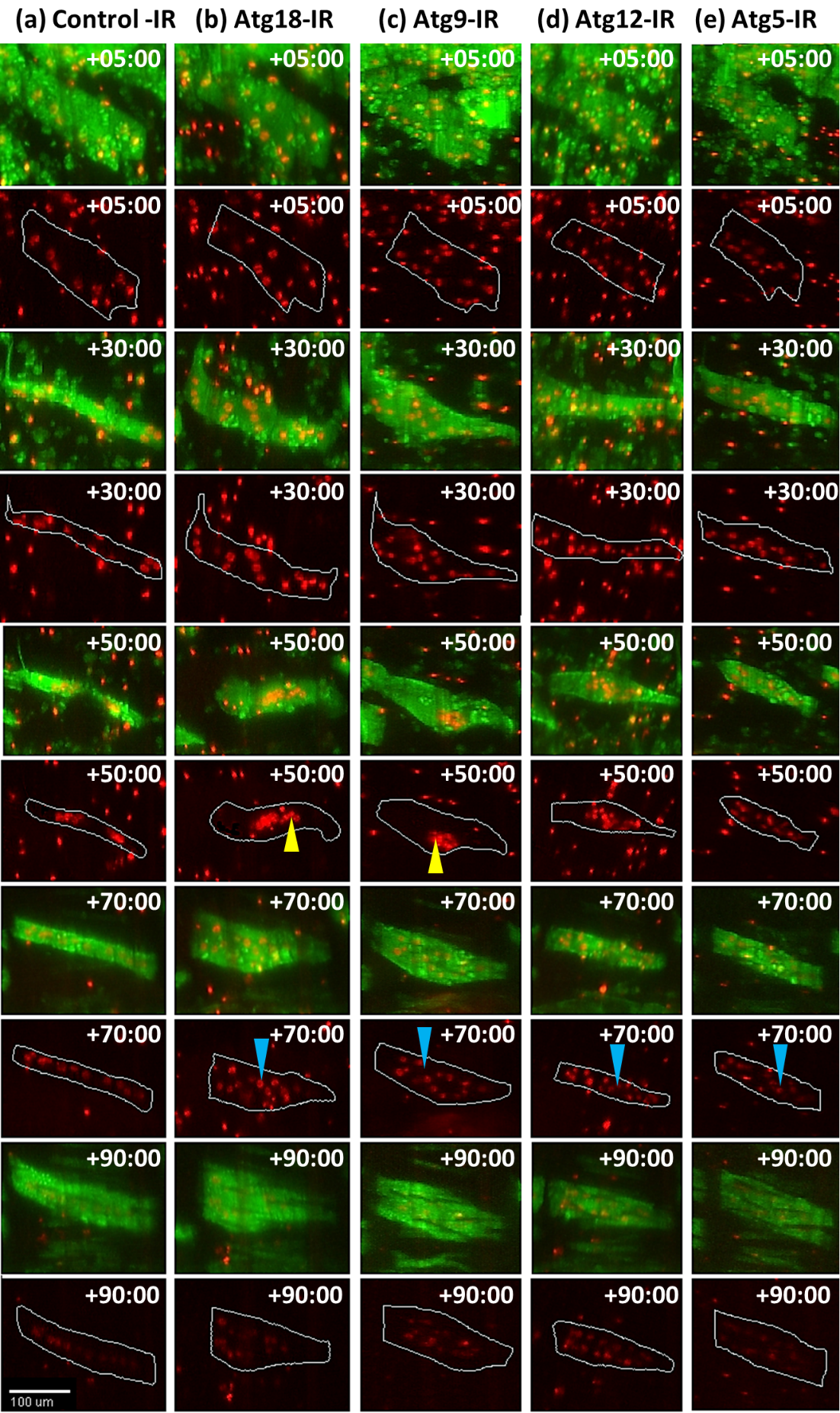

Supplement: Additional file 1: — Sample table of nuclear tracks, results of anti-polar/polar migration analysis and figures showing myonuclear distribution phenotypes observed in previous study. (DOCX 5608 kb) [file 12859_2017_1739_MOESM1_ESM.docx]
